# Bidirectional links between cyberchondria and health anxiety in Tunisian students

**DOI:** 10.1192/j.eurpsy.2026.10786

**Published:** 2026-07-31

**Authors:** H. Guidara, Z. Hadrich, I. Chaari, M. Kessentini, W. Abid, E. Maaloul, A. Kammoun, F. Charfeddine, L. Aribi, N. Messedi, J. Aloulou

**Affiliations:** 1 Psychiatric department “B”, Hedi Chaker University Hospital; 2 Higher institute of nursing sciences of Sfax, Sfax, Tunisia

## Abstract

**Introduction:**

Cyberchondria and health anxiety are intertwined. Online health searches can temporarily reassure but ultimately reinforce anxiety. Little is known about this dynamic in North African student populations.

**Objectives:**

To examine the association between cyberchondria and health anxiety in Tunisian students.

**Methods:**

We conducted a cross-sectional online survey among university students in Sfax (Tunisia) between March and April 2025. Participants completed Cyberchondria Severity Scale-15 (CSS-15) and Short form of Health Anxiety Inventory (SHAI). Analyses used non-parametric tests and Spearman correlation.

**Results:**

A total of 283 Tunisian students participated (58.7% were male, mean age 21.32 ± 1.41 years). Cyberchondria was moderate in 64% and high in 3.9%. Health anxiety was moderate to substantial in 16.2%. Cyberchondria correlated positively with health anxiety (ρ=0.325, p<0.001). Students with family psychiatric history had the highest combined scores. Females consistently reported higher severity (p<0.001).

**Conclusions:**

Among Tunisian students, cyberchondria and health anxiety reinforce each other, forming a vicious cycle of worry and online checking. Interventions focusing on psychoeducation and digital health awareness are warranted.

**Disclosure of Interest:**

None Declared

